# (*E*)-Methyl *N*′-(3,4,5-trimethoxy­benzyl­idene)hydrazinecarboxyl­ate

**DOI:** 10.1107/S1600536808031590

**Published:** 2008-10-04

**Authors:** Lu-Ping Lv, Jian-Wu Xie, Wen-Bo Yu, Wei-Wei Li, Xian-Chao Hu

**Affiliations:** a Department of Chemical Engineering, Hangzhou Vocational and Technical College, Hangzhou 310018, People’s Republic of China; b Research Center of Analysis and Measurement, Zhejiang University of Technology, Hangzhou 310014, People’s Republic of China

## Abstract

The mol­ecule of the title compound, C_12_H_16_N_2_O_5_, adopts a *trans* configuration with respect to the C=N double bond. The dihedral angle between the benzene and hydrazinecarboxylic acid methyl ester planes is 12.55 (7)°. The mol­ecules are linked into a chain along [001] by inter­molecular N—H⋯O hydrogen bonds, and the chains are cross-linked into a two-dimensional zigzag structure by C—H⋯O hydrogen bonds.

## Related literature

For general background, see: Parashar *et al.* (1988 ▶); Hadjoudis *et al.* (1987 ▶); Borg *et al.* (1999 ▶). For a related structure, see: Shang *et al.* (2007 ▶).
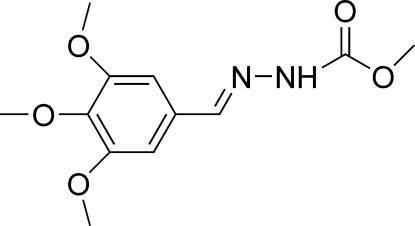


## Experimental

### 

#### Crystal data

C_12_H_16_N_2_O_5_
*M* *_r_* = 268.27
Monoclinic, 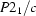

*a* = 8.554 (3) Å
*b* = 22.705 (7) Å
*c* = 7.813 (2) Å
β = 116.15 (1)°
*V* = 1362.1 (7) Å^3^
*Z* = 4
Mo *K*α radiation
μ = 0.10 mm^−1^
*T* = 273 (2) K
0.27 × 0.25 × 0.24 mm

#### Data collection

Bruker SMART CCD area-detector diffractometer
Absorption correction: multi-scan (*SADABS*; Bruker, 2002 ▶) *T*_min_ = 0.965, *T*_max_ = 0.968
7173 measured reflections
2394 independent reflections
1671 reflections with *I* > 2σ(*I*)
*R*_int_ = 0.058

#### Refinement

*R*[*F* ^2^ > 2σ(*F* ^2^)] = 0.044
*wR*(*F* ^2^) = 0.133
*S* = 1.03
2394 reflections
177 parameters
H-atom parameters constrained
Δρ_max_ = 0.18 e Å^−3^
Δρ_min_ = −0.18 e Å^−3^

### 

Data collection: *SMART* (Bruker, 2002 ▶); cell refinement: *SAINT* (Bruker, 2002 ▶); data reduction: *SAINT*; program(s) used to solve structure: *SHELXS97* (Sheldrick, 2008 ▶); program(s) used to refine structure: *SHELXL97* (Sheldrick, 2008 ▶); molecular graphics: *SHELXTL* (Sheldrick, 2008 ▶); software used to prepare material for publication: *SHELXTL*.

## Supplementary Material

Crystal structure: contains datablocks I, global. DOI: 10.1107/S1600536808031590/ci2687sup1.cif

Structure factors: contains datablocks I. DOI: 10.1107/S1600536808031590/ci2687Isup2.hkl

Additional supplementary materials:  crystallographic information; 3D view; checkCIF report

## Figures and Tables

**Table 1:** Hydrogen-bond geometry (Å, °)

*D*—H⋯*A*	*D*—H	H⋯*A*	*D*⋯*A*	*D*—H⋯*A*
N2—H2⋯O4^i^	0.86	2.16	3.000 (2)	166
C2—H2*B*⋯O2^ii^	0.96	2.57	3.498 (3)	161

Symmetry codes: (i) 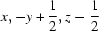; (ii) 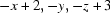.

**Fig. 1.**
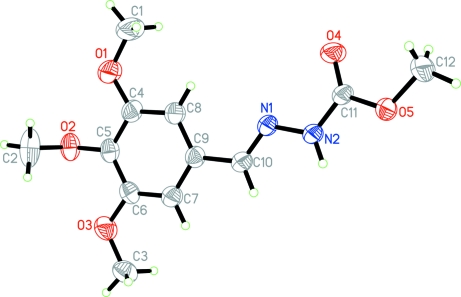
The molecular structure of the title compound, showing 40% probability displacement ellipsoids and the atomic numbering.

**Fig. 2.**
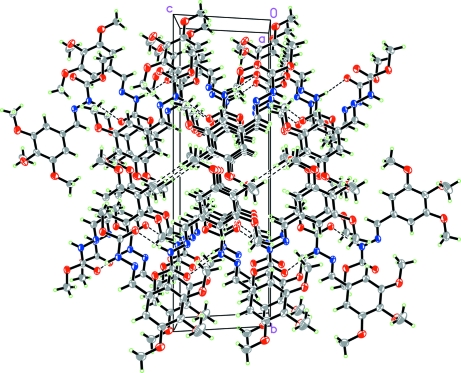
Crystal packing of the title compound, viewed approximately down the a axis. Dashed lines indicate hydrogen bonds.

## supplementary crystallographic information

### Comment

Benzaldehydehydrazone derivatives have received considerable attention for a long time due to their pharmacological activity (Parashar *et al.*, 1988) and their photochromic properties(Hadjoudis *et al.*, 1987). They are important intermidiates of 1,3,4-oxadiazoles, which have been reported to be versatile compounds with many properties (Borg *et al.*, 1999). As a further investigation of this type of derivatives, we report herein the crystal structure of the title compound.

The title molecule (Fig.1) adopts a trans configuration with respect to the C═N bond. The hydrazine carboxylic acid methyl ester group is slightly twisted away from the attached ring. The dihedral angle between the benzene ring and the C10/C11//N1/N2/O4/O5 plane [r.m.s. deviation 0.051 Å] is 12.55 (7)°. The O1-C1 and O3-C3 methoxy groups are coplanar with the benzene ring [C8—C4—O1—C1 = -1.7 (3)° and C7—C6—O3—C3 = -1.9 (3)°] while the O2-C2 group is twisted almost perpendicular to the attached ring [C6—C5—O2—C2 = 91.6 (2)°]. The bond lengths and angles agree with those observed for N'-(4-methoxybenzylidene)methoxyformohydrazide (Shang *et al.*, 2007).

The molecules are linked into a chain along the [001] by intermolecular N–H···O hydrogen bonds (Fig.2 and Table 1). The chains are cross-linked into a two-dimensional zigzag structure by C—H···O hydrogen bonds.

### Experimental

3,4,5-Trimethoxybenzaldehyde (1.96g, 0.01mol) and methyl hydrazinecarboxylate (0.9 g, 0.01 mol) were dissolved in stirred methanol (15 ml) and left for 3.2 h at room temperature. The resulting solid was filtered off and recrystallized from ethanol to give the title compound in 94% yield. Single crystals suitable for X-ray analysis were obtained by slow evaporation of an ethanol solution at room temperature (m.p. 472-474 K).

### Refinement

H atoms were positioned geometrically [N-H = 0.86 Å and C-H = 0.93 or 0.96 Å] and refined using a riding model, with *U*_iso_(H) = 1.2*U*_eq_(C,N) and 1.5U_eq_(C_methyl_). A rotating group model was used for the methy groups.

### Crystal data

**Table:**

C_12_H_16_N_2_O_5_	*F*(000) = 568
*M**_r_* = 268.27	*D*_x_ = 1.308 Mg m^−^^3^
Monoclinic, *P*2_1_/*c*	Mo *K*α radiation, λ = 0.71073 Å
Hall symbol: -P 2ybc	Cell parameters from 2394 reflections
*a* = 8.554 (3) Å	θ = 1.8–25.0°
*b* = 22.705 (7) Å	µ = 0.10 mm^−^^1^
*c* = 7.813 (2) Å	*T* = 273 K
β = 116.15 (1)°	Block, colourless
*V* = 1362.1 (7) Å^3^	0.27 × 0.25 × 0.24 mm
*Z* = 4	

### Data collection

**Table:**

Bruker SMART CCD area-detector diffractometer	2394 independent reflections
Radiation source: fine-focus sealed tube	1671 reflections with *I* > 2σ(*I*)
graphite	*R*_int_ = 0.058
φ and ω scans	θ_max_ = 25.1°, θ_min_ = 1.8°
Absorption correction: multi-scan (SADABS; Bruker, 2002)	*h* = −10→10
*T*_min_ = 0.965, *T*_max_ = 0.968	*k* = −27→26
7173 measured reflections	*l* = −9→9

### Refinement

**Table:**

Refinement on *F*^2^	Secondary atom site location: difference Fourier map
Least-squares matrix: full	Hydrogen site location: inferred from neighbouring sites
*R*[*F*^2^ > 2σ(*F*^2^)] = 0.044	H-atom parameters constrained
*wR*(*F*^2^) = 0.133	*w* = 1/[σ^2^(*F*_o_^2^) + (0.0696*P*)^2^] where *P* = (*F*_o_^2^ + 2*F*_c_^2^)/3
*S* = 1.03	(Δ/σ)_max_ = 0.001
2394 reflections	Δρ_max_ = 0.18 e Å^−^^3^
177 parameters	Δρ_min_ = −0.18 e Å^−^^3^
0 restraints	Extinction correction: SHELXL97 (Sheldrick, 2008), Fc^*^=kFc[1+0.001xFc^2^λ^3^/sin(2θ)]^-1/4^
Primary atom site location: structure-invariant direct methods	Extinction coefficient: 0.012 (3)

### Special details

**Table:**

Geometry. All esds (except the esd in the dihedral angle between two l.s. planes) are estimated using the full covariance matrix. The cell esds are taken into account individually in the estimation of esds in distances, angles and torsion angles; correlations between esds in cell parameters are only used when they are defined by crystal symmetry. An approximate (isotropic) treatment of cell esds is used for estimating esds involving l.s. planes.
Refinement. Refinement of F^2^ against ALL reflections. The weighted R-factor wR and goodness of fit S are based on F^2^, conventional R-factors R are based on F, with F set to zero for negative F^2^. The threshold expression of F^2^ > 2sigma(F^2^) is used only for calculating R-factors(gt) etc. and is not relevant to the choice of reflections for refinement. R-factors based on F^2^ are statistically about twice as large as those based on F, and R- factors based on ALL data will be even larger.

### Fractional atomic coordinates and isotropic or equivalent isotropic displacement parameters (Å^2^)

**Table:**

	*x*	*y*	*z*	*U*_iso_*/*U*_eq_	
C11	0.1250 (2)	0.29913 (8)	0.4985 (2)	0.0442 (5)	
C7	0.5205 (3)	0.07771 (8)	0.7843 (3)	0.0522 (5)	
H7	0.4723	0.0607	0.6636	0.063*	
C10	0.3534 (3)	0.16740 (8)	0.6404 (3)	0.0493 (5)	
H10	0.3171	0.1508	0.5201	0.059*	
C9	0.4743 (2)	0.13484 (8)	0.8080 (2)	0.0467 (5)	
C6	0.6382 (3)	0.04596 (8)	0.9394 (3)	0.0508 (5)	
C8	0.5451 (3)	0.16011 (9)	0.9895 (2)	0.0529 (5)	
H8	0.5130	0.1980	1.0067	0.063*	
C4	0.6637 (3)	0.12845 (9)	1.1436 (3)	0.0526 (5)	
C5	0.7116 (2)	0.07116 (8)	1.1194 (3)	0.0499 (5)	
C12	−0.0302 (4)	0.37985 (10)	0.3123 (3)	0.0850 (8)	
H12A	−0.0984	0.3825	0.3820	0.127*	
H12B	−0.1010	0.3900	0.1813	0.127*	
H12C	0.0666	0.4065	0.3662	0.127*	
C3	0.6171 (3)	−0.03833 (9)	0.7485 (3)	0.0677 (6)	
H3A	0.4927	−0.0390	0.7001	0.102*	
H3B	0.6600	−0.0779	0.7609	0.102*	
H3C	0.6482	−0.0169	0.6620	0.102*	
C1	0.7014 (4)	0.20690 (11)	1.3617 (3)	0.0986 (10)	
H1A	0.7253	0.2340	1.2818	0.148*	
H1B	0.7713	0.2169	1.4931	0.148*	
H1C	0.5804	0.2092	1.3335	0.148*	
C2	1.0022 (3)	0.04729 (13)	1.3133 (4)	0.0950 (9)	
H2A	1.0190	0.0332	1.2067	0.143*	
H2B	1.0752	0.0254	1.4253	0.143*	
H2C	1.0323	0.0883	1.3335	0.143*	
O5	0.03345 (18)	0.32059 (5)	0.32345 (16)	0.0574 (4)	
O4	0.1466 (2)	0.32391 (5)	0.64414 (17)	0.0632 (5)	
O2	0.82545 (17)	0.03987 (6)	1.27554 (19)	0.0608 (4)	
O3	0.6917 (2)	−0.01046 (6)	0.9293 (2)	0.0670 (5)	
O1	0.7416 (2)	0.14863 (6)	1.32715 (18)	0.0745 (5)	
N1	0.29649 (19)	0.21810 (6)	0.65536 (19)	0.0442 (4)	
N2	0.1871 (2)	0.24529 (6)	0.4883 (2)	0.0492 (4)	
H2	0.1588	0.2285	0.3801	0.059*	

### Atomic displacement parameters (Å^2^)

**Table:**

	*U*^11^	*U*^22^	*U*^33^	*U*^12^	*U*^13^	*U*^23^
C11	0.0513 (12)	0.0478 (10)	0.0312 (9)	0.0013 (9)	0.0160 (8)	−0.0003 (8)
C7	0.0572 (13)	0.0525 (11)	0.0440 (11)	0.0038 (9)	0.0198 (10)	0.0014 (8)
C10	0.0541 (13)	0.0511 (11)	0.0382 (10)	0.0028 (9)	0.0162 (9)	−0.0023 (8)
C9	0.0474 (11)	0.0509 (11)	0.0403 (10)	0.0040 (8)	0.0180 (9)	0.0054 (8)
C6	0.0510 (12)	0.0468 (11)	0.0576 (12)	0.0075 (9)	0.0268 (10)	0.0085 (9)
C8	0.0588 (13)	0.0498 (10)	0.0445 (11)	0.0071 (10)	0.0178 (10)	0.0048 (8)
C4	0.0564 (13)	0.0591 (12)	0.0382 (10)	0.0032 (10)	0.0171 (9)	0.0068 (8)
C5	0.0466 (11)	0.0554 (11)	0.0478 (11)	0.0073 (9)	0.0211 (9)	0.0165 (9)
C12	0.118 (2)	0.0590 (14)	0.0569 (14)	0.0348 (14)	0.0188 (14)	0.0078 (10)
C3	0.0801 (17)	0.0546 (12)	0.0745 (16)	0.0101 (11)	0.0395 (13)	0.0015 (10)
C1	0.134 (3)	0.0820 (17)	0.0484 (13)	0.0263 (17)	0.0111 (15)	−0.0086 (11)
C2	0.0506 (16)	0.118 (2)	0.101 (2)	0.0070 (14)	0.0191 (14)	0.0537 (17)
O5	0.0733 (10)	0.0556 (8)	0.0359 (7)	0.0213 (7)	0.0174 (7)	0.0050 (5)
O4	0.0951 (12)	0.0523 (8)	0.0379 (8)	0.0124 (7)	0.0253 (7)	−0.0012 (6)
O2	0.0523 (9)	0.0694 (9)	0.0573 (9)	0.0100 (7)	0.0211 (7)	0.0266 (7)
O3	0.0761 (10)	0.0544 (9)	0.0661 (10)	0.0191 (7)	0.0275 (8)	0.0098 (7)
O1	0.0913 (13)	0.0709 (10)	0.0415 (8)	0.0192 (8)	0.0111 (8)	0.0042 (7)
N1	0.0497 (10)	0.0482 (9)	0.0317 (8)	0.0036 (7)	0.0152 (7)	0.0034 (6)
N2	0.0616 (11)	0.0501 (9)	0.0296 (7)	0.0133 (8)	0.0145 (7)	0.0004 (6)

### Geometric parameters (Å, °)

**Table:**

C11—O4	1.209 (2)	C12—H12A	0.96
C11—O5	1.333 (2)	C12—H12B	0.96
C11—N2	1.349 (2)	C12—H12C	0.96
C7—C6	1.388 (2)	C3—O3	1.417 (2)
C7—C9	1.392 (3)	C3—H3A	0.96
C7—H7	0.93	C3—H3B	0.96
C10—N1	1.275 (2)	C3—H3C	0.96
C10—C9	1.462 (2)	C1—O1	1.422 (3)
C10—H10	0.93	C1—H1A	0.96
C9—C8	1.396 (2)	C1—H1B	0.96
C6—O3	1.374 (2)	C1—H1C	0.96
C6—C5	1.386 (3)	C2—O2	1.417 (3)
C8—C4	1.386 (2)	C2—H2A	0.96
C8—H8	0.93	C2—H2B	0.96
C4—O1	1.367 (2)	C2—H2C	0.96
C4—C5	1.401 (3)	N1—N2	1.3723 (19)
C5—O2	1.376 (2)	N2—H2	0.86
C12—O5	1.439 (2)		
			
O4—C11—O5	124.93 (17)	H12A—C12—H12C	109.5
O4—C11—N2	125.21 (16)	H12B—C12—H12C	109.5
O5—C11—N2	109.85 (14)	O3—C3—H3A	109.5
C6—C7—C9	120.45 (17)	O3—C3—H3B	109.5
C6—C7—H7	119.8	H3A—C3—H3B	109.5
C9—C7—H7	119.8	O3—C3—H3C	109.5
N1—C10—C9	121.47 (17)	H3A—C3—H3C	109.5
N1—C10—H10	119.3	H3B—C3—H3C	109.5
C9—C10—H10	119.3	O1—C1—H1A	109.5
C7—C9—C8	119.77 (17)	O1—C1—H1B	109.5
C7—C9—C10	118.81 (16)	H1A—C1—H1B	109.5
C8—C9—C10	121.41 (17)	O1—C1—H1C	109.5
O3—C6—C5	115.43 (16)	H1A—C1—H1C	109.5
O3—C6—C7	124.47 (17)	H1B—C1—H1C	109.5
C5—C6—C7	120.11 (17)	O2—C2—H2A	109.5
C4—C8—C9	119.57 (18)	O2—C2—H2B	109.5
C4—C8—H8	120.2	H2A—C2—H2B	109.5
C9—C8—H8	120.2	O2—C2—H2C	109.5
O1—C4—C8	124.72 (18)	H2A—C2—H2C	109.5
O1—C4—C5	114.63 (16)	H2B—C2—H2C	109.5
C8—C4—C5	120.65 (17)	C11—O5—C12	116.06 (14)
O2—C5—C6	120.91 (17)	C5—O2—C2	113.36 (15)
O2—C5—C4	119.61 (17)	C6—O3—C3	117.27 (15)
C6—C5—C4	119.44 (16)	C4—O1—C1	117.67 (16)
O5—C12—H12A	109.5	C10—N1—N2	116.53 (14)
O5—C12—H12B	109.5	C11—N2—N1	118.23 (14)
H12A—C12—H12B	109.5	C11—N2—H2	120.9
O5—C12—H12C	109.5	N1—N2—H2	120.9
			
C6—C7—C9—C8	−0.7 (3)	C8—C4—C5—O2	−177.98 (18)
C6—C7—C9—C10	178.45 (18)	O1—C4—C5—C6	178.96 (17)
N1—C10—C9—C7	174.74 (18)	C8—C4—C5—C6	−0.5 (3)
N1—C10—C9—C8	−6.1 (3)	O4—C11—O5—C12	5.6 (3)
C9—C7—C6—O3	−179.79 (18)	N2—C11—O5—C12	−175.51 (18)
C9—C7—C6—C5	−0.5 (3)	C6—C5—O2—C2	91.6 (2)
C7—C9—C8—C4	1.3 (3)	C4—C5—O2—C2	−91.0 (2)
C10—C9—C8—C4	−177.86 (18)	C5—C6—O3—C3	178.74 (18)
C9—C8—C4—O1	179.89 (18)	C7—C6—O3—C3	−1.9 (3)
C9—C8—C4—C5	−0.7 (3)	C8—C4—O1—C1	−1.7 (3)
O3—C6—C5—O2	−2.1 (3)	C5—C4—O1—C1	178.9 (2)
C7—C6—C5—O2	178.52 (17)	C9—C10—N1—N2	178.42 (17)
O3—C6—C5—C4	−179.53 (17)	O4—C11—N2—N1	−6.2 (3)
C7—C6—C5—C4	1.1 (3)	O5—C11—N2—N1	174.90 (15)
O1—C4—C5—O2	1.5 (3)	C10—N1—N2—C11	−179.66 (17)

### Hydrogen-bond geometry (Å, °)

**Table:**

*D*—H···*A*	*D*—H	H···*A*	*D*···*A*	*D*—H···*A*
N2—H2···O4^i^	0.86	2.16	3.000 (2)	166
C2—H2B···O2^ii^	0.96	2.57	3.498 (3)	161

Symmetry codes: (i) *x*, −*y*+1/2, *z*−1/2; (ii) −*x*+2, −*y*, −*z*+3.

## References

[bb1] Borg, S., Vollinga, R. C., Labarre, M., Payza, K., Terenius, L. & Luthman, K. (1999). *J. Med. Chem.* **42**, 4331–4342. 10.1021/jm990197+ 10543877

[bb2] Bruker (2002). *SMART*, *SAINT* and *SADABS* Bruker AXS Inc., Madison, Wisconsin, USA.

[bb3] Hadjoudis, E., Vittorakis, M. & Moustakali-Mavridis, J. (1987). *Tetrahedron*, **43**, 1345–1360.

[bb4] Parashar, R. K., Sharma, R. C., Kumar, A. & Mohanm, G. (1988). *Inorg. Chim Acta*, **151**, 201–208.

[bb5] Shang, Z.-H., Zhang, H.-L. & Ding, Y. (2007). *Acta Cryst.* E**63**, o3394.

[bb6] Sheldrick, G. M. (2008). *Acta Cryst.* A**64**, 112–122. 10.1107/S0108767307043930 18156677

